# Comparison of published orthopaedic trauma trials following registration in Clinicaltrials.gov

**DOI:** 10.1186/1471-2474-12-278

**Published:** 2011-12-07

**Authors:** Rajiv Gandhi, Meryam Jan, Holly N Smith, Nizar N Mahomed, Mohit Bhandari

**Affiliations:** 1 Toronto Western Hospital EW 1-439, 399 Bathurst Street, Toronto, ON M5T 2S8, Canada; 2 Toronto Western Hospital EW 1-433, 399 Bathurst Street, Toronto, ON M5T 2S8, Canada; 3 Toronto Western Hospital EW 1-435, 399 Bathurst Street, Toronto, ON M5T 2S8, Canada; 4 Hamilton General Hospital, 7 North, Suite 727 237 Barton St East, Hamilton, ON L8L 2X2, Canada

**Keywords:** Trial registration, Clinicaltrials.gov, Orthopaedic trauma

## Abstract

**Background:**

After the Food and Drug Administration Modernization Act of 1997, the registration of all clinical trials became mandatory prior to publication. Our primary objective was to determine publication rates for orthopaedic trauma trials registered with ClinicalTrials.gov. We further evaluated methodological consistency between registration and publication.

**Methods:**

We searched Clinical Trials.gov for all trials related to orthopaedic trauma. We excluded active trials and trials not completed by July 2009, and performed a systematic search for publications resulting from registered closed trials. Information regarding primary and secondary outcomes, intervention, study sponsors, and sample size were extracted from registrations and publications.

**Results:**

Of 130 closed trials, 37 eligible trials resulted in 16 publications (43.2%). We found no significant differences in publication rates between funding sources for industry sponsored studies and nongovernment/nonindustry sponsored studies (*p *> 0.05). About half the trials (45%) did not include the NCT ID in the publication. Two (10%) publications had major changes to the primary outcome measure and ten (52.6%) to sample size.

**Conclusions:**

Registration of orthopaedic trauma trials does not consistently result in publication. When trials are registered, many do not cite NCT ID in the publication. Furthermore, changes that are not reflected in the registry of the trial are frequently made to the final publication.

## Introduction

ClinicalTrials.gov (CTG) was developed by the U.S. National Institutes of Health in collaboration with the Food and Drug Administration (FDA) in February 2000 [1]. It was established after the FDA Modernization Act of 1997, which made it a requirement for the Department of Health and Human Services to establish a registry of all clinical trials of experimental treatments for serious or life-threatening diseases or conditions [2]. It offers current information on clinical trials for a wide range of diseases and conditions, allowing doctors, researchers, and patients to locate clinical trials conducted worldwide. CTG currently contains over 94,000 registered trials from 174 countries [3].

The database summarizes each registered trial and gives information including but not limited to the purpose of the study, recruiting status, criteria for patient participation, and location of the trial and specific contact information. Furthermore, additional information such as research study design and condition under study is outlined to help potential participants consider a particular trial.

Each listing on the registry is given a unique National Clinical Trials Identifier (NCT ID) with the prefix 'NCT' and an 8-digit number that is used to locate and identify a specific trial. We believe that the NCT ID should be included in any final publication to allow the reader to evaluate the strength of the trial by comparing it to the original plans as outlined in the registry. We performed a review of trials registered in CTG and relating to orthopaedic trauma evaluating publication rates and consistency of reporting. We hypothesized that the incidence of registered trials not published would be high despite mandatory reporting of clinical trials, and there would be methodological differences between publication and registration methodology.

## Methods

### Eligibility criteria

Trials that were considered eligible to be included in our analysis met the following criteria: 1) registered on clinicaltrials.gov registry, 2) registered up to July 2009, and 3) trials that were reported as closed. Ongoing trials were excluded from analysis.

### Search strategy

We performed a search of orthopaedic trauma trials with the http://www.ClinicalTrials.gov database in duplicate (MJ, HS) using the search terms (*orthopaedic trauma *OR *orthopedic trauma*). We also performed a search using the conditions listed on CTG including "wounds and injuries" and "fracture, bone," "radius fracture," "tibia fracture" and "sprains and strains." This resulted in 264 registered trials up to and including July 2010. The search was further narrowed to closed studies with the understanding that studies still actively recruiting participants are unlikely to have publications (n = 134). Refining the search resulted in 130 closed studies. Of these, we decided to review studies with an estimated completion date up to and including July 2009 to allow adequate time for preparation and submission of a manuscript and therefore excluded those trials with a completion date after July 2009 (n = 67). Seven studies did not indicate a completion date or estimated completion date in the database and were thus excluded. We also excluded non-randomized and observational studies. Application of this criteria resulted in 37 studies for subsequent analysis. Discrepancies in eligibility were resolved by consensus. Agreement for final inclusion between reviewers was excellent. (Kappa = 0.79; 95% confidence interval, 0.46 to 0.94).

Because our results from our previous search terms provided us with a larger number of trials to analyze, we believe our search strategy is sufficient.

### Publication rate

Each of the 130 closed studies were first assessed to determine whether or not the investigators listed any publications within the trial registry. If none were listed, a search using key terms from the trial and the primary investigator was performed using the Pubmed, EMBASE and Ovid Medline databases. Suspected publications were confirmed with the registry based on NCT ID, publication date, authors, number of participants, primary and secondary outcome measures, and study location.

### Data abstraction

After identifying a published study, two of us (HS, MJ) extracted data from both the CTG database and the publication including authors, year of publication, NCT ID and its presence in the paper, trial status (complete, active, not recruiting etc.), study start date, registration date and completion date, study sponsor, primary and secondary outcome measures, study phase and design, study location, inclusion and exclusion criteria, and sample size.

We evaluated the agreement between the methodology reported in the registry and that of the publication. Major discrepancies were defined using a modification of criteria previously reported by Chan et al. [4]: (1) a pre-specified primary outcome was reported as secondary or was not labeled as either primary or secondary in the publication; (2) a pre-specified primary outcome was omitted from the published article; (3) a new primary outcome was introduced in the published article.

### Statistical analysis

Study characteristics were presented with descriptive statistics. Association between trial sponsor and incidence of publication was anlayzed with the Fisher exact test. All p values were two tailed with a level of significance set at 0.05.

Kappa statistics were used to calculate the agreement between reviewers on study eligibility.

## Results

### Characteristics of registered trials

The 37 eligible orthopaedic trauma trials included in CTG spanned North and South America, Europe, Asia and Africa. Most trials evaluated surgical interventions, however there were also drug and therapeutic interventions such as the use radiation. Further details of the eligible trials can be found in Table 1.

**Table 1:** Details of Eligible Trials

	Orthopaedic Trauma
Number of Trials	36

Number of Publications (%)	20 (54%)

Intervention	

Surgical	20

Drug	14

Other	3

Median Estimated Sample Size	298

(Range)	(22-3432)

Sponsor	

Industry	14

Government	4

Nonindustry/nongovernment	19

Characteristics of the 37 closed trials identified in CTG that were completed up to and including July 2009.

### Publication rate

In total, 16 of 37 (43.2%) registered trials with an estimated completion date up to and including July 2009 were confirmed to have publications. Four interim publications [5-8] defined as ongoing trials with publications prior to the study completion were also identified for a total of 20 publications included in our analysis of consistency with trial registration. Of the 4 interim publications, 1 was sponsored by industry [7], and the remaining 3 by a nongovernment/nonindustry source [5,6,8]. Nongovernment/nonindustry sponsored trials had the highest publication rate, with 9 of 19 (47.3%) trials resulted in publication [9-17]. In comparison, 6 of 14 (42.9%) industry sponsored trials resulted in publication [18-23], and 1 of 4 (25%) government sponsored trials [24] were published. There were no significant differences in publication rates for industry sponsored studies and nongovernment/nonindustry sponsored studies (*p *= 0.92), between government sponsored and nongovernment/nonindustry sponsored trials (*p *= 0.6) or industry sponsored trials (*p *= 0.62).

Fifteen of the 20 publications were from a trial with a status of "completed" in CTG [9-19,21-24]. The remaining publications were listed as active but not recruiting [5-8], and terminated in the database [20]. One additional study had a published abstract but no complete manuscript [25]. We verified this by confirming that the title, author, outcome measures, and study dates were the same as those mentioned in the registered trial. Of the 20 publications, the NCT ID was included in 11 (55%) [8,9,11,12,14,15,17,18,20,23,24].

Characteristics of published trials can be found in Table 2.

**Table 2:** Characteristics of Published Orthopaedic Trauma Trials

	No. (%) of Articles		
	**Industry**	**Government**	**Neither**

	**(n = 14)**	**(n = 4)**	**(n = 19)**

Number of Registered Trials	14/37	4/37	19/37

Trials Published	6	1	9

Interim Publications	1	0	3

NCT ID Reported in Publication (%)	3 (42.8)	1 (100)	7 (58.3)

Major Outcome Measure Discrepancies	1 (14.2)	0	3 (25)

Primary Outcome Not Labelled in	1	0	1

Publication			

Secondary Outcome Not Labelled in	0	0	2

Publication			

Discrepancies Favouring Statistically	0	0	0

Significant Results			

Major Sample Size Changes (%)*	3 (42.8)	0	6 (50)

Trial Type			

Interventional RCT	6 (100)	1 (100)	9 (100)

Characteristics of trials conducted in North America, Europe and New Zealand registered on CTG, classified by funding source

*defined as ± 6% of the sample size listed on CTG

### Consistency between trial registration and publication

We compared the study sample size proposed on CTG with the actual study enrolment reported in the publication. There was one study that did not include a proposed sample size in the registry [10]. In 9 of the remaining 19 studies (47.3%), enrolment as stated in CTG exactly matched that of the final publication [5,7-9,12,16-18,24]. Of the 10 publications (52.6%) with an inconsistent sample size, 7 had a smaller sample than the one reported in CTG [6,11,13,15,19,20,22], and 3 had a larger sample [14,21,23]. The sample size discrepancy in all publications differed from the original figure by a minimum of ± 6%.

We compared the agreement between the primary and secondary outcome measures as stated in the publication and on CTG. The registry listed a primary outcome measure for all trials and secondary outcome measures for 18 trials. One trial did not list a secondary outcome measure in the registry or publication [24]. Two of the 20 publications (10%) had a major discrepancy with the registry. Each of these publications did not state the primary outcome measure despite identifying one in the registry [6,18]. All but two of the remaining 18 publications were consistent with the registry with regards to primary and secondary outcomes [5,7-12,15-17,19-23]. Two publications did not report a secondary outcome measure in the manuscript that had been listed in the registry [13,14].

## Discussion

As a result of our search of orthopaedic trauma trials registered in CTG, we found that only 16 of 37 (43.2%) trials resulted in publication as of March 2011. Furthermore, final results of completed trials were not reported in the registry, and many publications were inconsistent in sample size and reporting of outcomes with the original report in CTG. We are unable to determine the reason for trials failing to result in publication. Several possibilities exist, including that the data was never analyzed, the journals elected not to publish, the authors elected not to publish the findings because they failed to substantiate the study hypothesis or believed the results contradicted the hypothesis. Another possibility may be that papers were not accepted for publication because of non-significant results. Regardless, the results of this study represent a large potential for publication bias in the orthopaedic trauma literature.

Historically, studies with statistically significant outcomes have been more likely to be submitted for publication and accepted by journals. Dickersinet al. [26] found that trials with statistically significant results were 2.9 times more likely to be published. Several authors have recently reported similar problems in the orthopaedic literature [27,28]. Publication bias may lead to wasted resources due to unnecessary duplicate studies, as well as potential harm to study participants [29].

Nongovernment/nonindustry sponsored studies demonstrated the highest rate of publication (47.3%), while industry sponsored trials were similar (42.9%). Only one of four (25%) completed government sponsored trials were published. This is contrary to the results of several studies that have found that in general, industry sponsored studies have the lowest rates of publication [30].

Almost half (45%) of trials did not include the NCT ID in their publication. Including the NCT ID in the publication should be made mandatory as it allows the reader to locate and identify the trial on CTG and compare the original study design with the published design. Published manuscripts where the power or primary outcome measures have been changed can alert the reader to concerns of study validity.

We also found that 2 (10%) publications had major changes to the primary outcome measure and 10 (52.6%) to sample size. Discrepancies between trial registry and publication have been observed in previous studies and are a concern as they may compromise the integrity of the conclusions that can be drawn from the study [31]. Simply adding patients to a study may take a clinically insignificant finding, and make it statistically significant for the reader. Mandatory registration would allow journal editors to examine trial information when reviewing the manuscript to uncover such discrepancies.

We reviewed requirements for authors in various orthopaedic surgery journals to verify whether the journals support trial registration prior to a publication. We found that the Journal of Arthroplasty, British Journal of Bone and Joint Surgery, Journal of Orthopaedic Surgery, and the Journal of Orthopaedic Trauma did not include information about requirements related to the registration of clinical trials. Acta Orthopaedica Scandinavica instructs authors to comply with the CONSORT guidelines for reporting of clinical trials [32]. Although these guidelines ensure that the author submits the registration number and the name of the registry in the publication, they do not require the author to register a clinical trial in a registry. Clinics in Orthopaedic Surgery, the American Journal of Bone and Joint Surgery, and BioMed Central explicitly stated that registration of all clinical trials was required. Improving instructions for authors submitting manuscripts to journals, and making trial registration a necessity is an essential step to move towards reliable reporting of clinical trial results [33,34].

The limitations of our study include the uncertainty of the time it may take between submission of a paper to a journal and approval for publication. A potential weakness is that although we reviewed studies until March 2011 (publication time between July 2009-March 2011), it is possible that there are completed trials in print that are currently awaiting publication. Some journals may publish studies at a faster pace than others. It may be that there were studies that were published without our knowledge, completed early, or submitted before their completion date. Furthermore, it may be that changes to trial information on CTG simply may not be updated frequently enough and may cause the available information to be misleading. Also, it is possible that studies that did not focus on orthpaedic trauma but included some aspect of it were overlooked because they did not correspond with the search terms on CTG.

Our study also has several strengths. We conducted an extensive search in duplicate for publications of closed studies using several different search methods. We also conducted exhaustive searches for interim publications, and publications resulting from studies listed as terminated or withdrawn.

## Conclusions

The results of our review of orthopedic trauma trials on ClinicalTrials.gov indicate that registration does not consistently result in publication or disclosure of results. Although only 10% of the publications had major discrepancy between trial registry and publication, smaller changes are frequently made to the final presentation of the data that are not reflected in the registry of the trial. When trials are registered, a great number of them do not cite the registration number in the publication, making it impossible for the reader to evaluate the study conclusions in relation to the original plans for the trial. We suggest all journals should make registration of clinical trials mandatory for publication.

## List of Abbreviations

(CTG): ClinicalTrials.gov; (NCT ID): National Clinical Trials Identifier.

## Competing interests

The authors declare that they have no competing interests.

## Authors' contributions

RG designed the study, participated in analysis of the data, and helped to draft the manuscript. MJ participated in analysis of data and the writing and editing of the manuscript. HNS participated in analysis of the data and editing of the manuscript. NNM helped design the study. MB participated in analysis of the data and editing of the manuscript. All authors read and approved the final manuscript.

## Pre-publication history

The pre-publication history for this paper can be accessed here:

http://www.biomedcentral.com/1471-2474/12/278/prepub

## References

[B1] The Lister Hill national center for biomedical communications technical report: ClinicalTrials.gov; a report to the board of scientific counselors http://www.lhncbc.nlm.nih.gov/lhc/docs/reports/2005/tr2005003.pdf

[B2] Fact sheet: ClinicalTrials.gov http://www.nlm.nih.gov/pubs/factsheets/clintrial.html

[B3] About ClinicalTrials.gov http://clinicaltrials.gov/ct2/info/about

[B4] Chan AW Hróbjartsson A Haahr MT Gotzsche PC Altman DG Empirical evidence for selective reporting of outcomes in randomized trials: comparison of protocols to published articles JAMA 2004 291 20 2457 2465 10.1001/jama.291.20.2457 15161896

[B5] Stannard JP Volgas DA Stewart R McGwin G Jr Alonso JE Negative pressure wound therapy after severe open fractures: a prospective randomized study J Orthop Trauma 2009 23 8 552 557 10.1097/BOT.0b013e3181a2e2b6 19704269

[B6] Stannard JP Robinston JT Anderson ER McGwin G Jr Volgas DA Alonso JE Negative pressure wound therapy to treat hematomas and surgical incisions following high-energy trauma J Trauma 2006 60 1301 1306 10.1097/01.ta.0000195996.73186.2e 16766975

[B7] Saris DBF Vanlauwe J Victor J Haspl M Bohnsack M Fortems Y Vandekerckhove B Almqvist KF Claes T Handelberg F Lagae K Van der Bauwhede J Vandenneucker H Yang KGA Jelic M Verdonk R Veulemans N Bellemans J Luyten FP Characterized chondrocyte implantation results in better structural repair when treating symptomatic cartilage defects of the knee in a randomized controlled trial versus microfracture Am J Sport Med 2008 36 2 235 246 10.1177/0363546507311095 18202295

[B8] Colbert AP Markov MS Carlson N Gregory WL Carlson H Elmer JP Static magnetic field therapy for carpal tunnel syndrome: a feasibility study Arch Phys Med Rehabil 2010 91 1098 1104 10.1016/j.apmr.2010.02.013 20599049 PMC3018287

[B9] Nasell H Adami J Samnegard E Tonnesen H Ponzer S Effect of smoking cessation intervention on results of acute fracture surgery J Bone Joint Surg Am 2010 92 1335 1342 10.2106/JBJS.I.00627 20516308

[B10] Casati A Danelli G Baciarello M Corradi M Leone S Di Canni S Fanelli G A prospective, randomized comparison between ultrasound and nerve stimulation guidance for multiple injection axillary brachial plexus block Anesthesiology 2007 106 992 996 10.1097/01.anes.0000265159.55179.e1 17457131

[B11] Pike J Mulpuri K Metzger M Ng G Wells N Goetz T Blinded, prospective, randomized clinical trial comparing colar, dorsal, and custom thermoplastic splinting in treatment of acute mallet finger J Hand Surg 2010 35A 580 588 10.1016/j.jhsa.2010.01.005 20353859

[B12] Frihagen F Nordsletten L Madsen JE Hemiarthroplasty or internal fixation for intracapsular displaced femoral neck fractures: randomised controlled trial BMJ 2007 335 1251 1254 10.1136/bmj.39399.456551.25 18056740 PMC2137068

[B13] Hiemstra LA Heard SM Sasyniuk TM Buchko GL Reed JG Monteleone BJ Knee immobilization for pain control after a hamstring tendon anterior cruciate ligament reconstruction Am J Sport Med 2009 37 1 56 64 10.1177/0363546508322896 18801944

[B14] Hu S Dong HL Li YZ Luo ZJ Sun L Yang QZ Yang LF Xiong L Effects of remote ischemic preconditioning on biochemical markers and neurologic outcomes in patients undergoing elective cervical decompression surgery: a prospective randomized controlled trial J Neurosurg Anesthesiol 2010 22 1 46 52 10.1097/ANA.0b013e3181c572bd 19996767

[B15] Boonriong T Tangtrakulwanich B Glabglay P Nimmaanrat S Comparing etoricoxib and celecoxib for preemptive analgesia for acute postoperative pain in patients undergoing arthroscopic anterior cruciate ligament reconstruction: a randomized controlled trial BMC Musculoskel Dis 2010 11 246 250 10.1186/1471-2474-11-246 PMC2975651 20973952

[B16] Rasmussen S Christensen M Mathiesen I Simonson O Shockwave therapy for chronic Achilles tendinopathy: a double-blind, randomized clinical trial of efficacy Acta Orthop 2008 79 2 249 256 10.1080/17453670710015058 18484252

[B17] Salovaara K Tuppurainen M Karkkainen M Rikkonen T Sandini L Sirola J Honkanen R Alhava E Kroger H Effect of Vitamin D3 and calcium on fracture risk in 65- to 71-year- old women: a population-based 3-year randomized, controlled trial--the OSTPRE- FPS J Bone Miner Res 2010 25 7 1487 1795 10.1002/jbmr.48 20200964

[B18] Zollinger PE Tuinebreijer WE Kreis RW Breederveld RS Effect of vitamin C on frequency of reflex sympathetic dystrophy in wrist fractures: a randomised trial Lancet 1999 354 2025 2028 10.1016/S0140-6736(99)03059-7 10636366

[B19] Eriksson BI Dahl OE Lassen MR Ward DP Rothlein R Davis G Turpie AG Partial factor IXa inhibition with TTP889 for prevention of venous thromboembolism: an exploratory study J Thromb Haemost 2008 6 457 463 10.1111/j.1538-7836.2007.02872.x 18088349

[B20] Alarcon-Segovia D Tumlin JA Furie RA McKay JD Cardiel MH Strand V Bagin RG Linnik MD Hepburn B LJP 394 for the prevention of renal flare in patients with systemic lupus erythematosus Arthritis Rheum 2003 48 2 442 454 10.1002/art.10763 12571854

[B21] Witjes JA Popolo GD Marberger M Jonsson O Kaps HP Chapple CR A multicenter, double-blind, randomized, parallel group study comparing polyvinyl chloride and polyvinylchloride-free catheter materials J Urology 2009 182 2794 2798 10.1016/j.juro.2009.08.047 19837425

[B22] Lyles KW Colon-Emeric CS Magaziner JS Adachi JD Pieper CF Mautalen C Hyldstrup L Recknor C Nordsletten L Moore KA Lavecchia C Zhang J Mesenbrink P Hodgson PK Abrams K Orloff JJ Horowitz Z Eriksen EF Boonen S Zoledronic acid and clinical fractures and mortality after hip fracture N Engl J Med 2007 357 18 1799 809 10.1056/NEJMoa074941 17878149 PMC2324066

[B23] Bovend'Eerdt TJ Dawes H Sackley C Izadi H Wade DT An Integrated Motor Imagery Program to Improve Functional Task Performance in Neurorehabilitation: a single-blind randomized controlled trial Arch Phys Med Rehab 2010 91 6 939 946 10.1016/j.apmr.2010.03.008 20510987

[B24] The SPRINT Investigators Bhandari M Guyatt G Tornetta P Schemitsch E Swiontkowski M Sanders D Walter SD Study to prospectively evaluate reamed intramedually nails in patients with tibial fractures (S.P.R.I.N.T.): Study rationale and design BMC Musculoskel Dis 2008 9 91 10.1186/1471-2474-9-91 PMC2446397 18573205

[B25] Hunter DJ Gross K McCree PI Ling L Hirko K Zhang B Harvey W A randomized trial of realignment therapy for treatment of medial knee osteoarthritis Osteoarthr Cartilage 2009 17 s173

[B26] Dickersin K Min YI Publication bias: the problem that won't go away Ann N Y Acad Sci 1993 703 135 146 10.1111/j.1749-6632.1993.tb26343.x 8192291

[B27] Harris IA Mourad M Kadir A Solomon MJ Young JM Publication bias in abstracts presented to the annual meeting to the American Academy of Orthopaedic Surgeons J Orthop Surg 2007 15 1 62 6 10.1177/230949900701500114 17429120

[B28] Hasenboehler EA Choudhry IK Newman JT Smith WR Ziran BH Stahel PF Bias towards publishing positive results in orthopedic and general surgery: a patient safety issue? Patient Saf Surg 2007 1 4 10 10.1186/1754-9493-1-4 18271997 PMC2241774

[B29] McCray AT Ide NC Design and implementation of a national clinical trials registry J Am Med Inform Assn 2000 7 3 313 23 10.1136/jamia.2000.0070313 PMC61435 10833169

[B30] Ross JS Mulvey GK Hines EM Nissen SE Krumholz HM Trial publication after registration in ClinicalTrials.gov: a cross-sectional analysis PLoS Med 2009 6 9 1 9 10.1371/journal.pmed.1000144 PMC2728480 19901971

[B31] Zarin DA Tse T Ide NC Trial registration at ClinicalTrials.gov between May and October 2005 N Engl J Med 2005 353 2779 2787 10.1056/NEJMsa053234 16382064 PMC1568386

[B32] Hopewel S Clarke M Moher D Wager E Middleton P Altman DG Schulz KF The CONSORT Group CONSORT for reporting randomized controlled trials in journal and conference abstracts: explanation and elaboration PLoS Med 2008 5 1 e20 10.1371/journal.pmed.0050020 18215107 PMC2211558

[B33] Abaid LN Grimes DA Schulz KF Reducing publication bias of prospective clinical trials through trial registration Contraception 2007 76 339 341 10.1016/j.contraception.2007.06.013 17963856

[B34] Sterling T Publication decisions and their possible effects on inferences drawn from tests of significance or vice versa J Am Stat Assoc 1959 54 30 34 10.2307/2282137

